# Development of Slow-Release Fertilizers with Function of Water Retention Using Eco-Friendly Starch Hydrogels

**DOI:** 10.3390/molecules29204835

**Published:** 2024-10-12

**Authors:** Yue Song, Litao Ma, Qingfei Duan, Huifang Xie, Xinyi Dong, Huaran Zhang, Long Yu

**Affiliations:** 1 Institute of Chemistry, Henan Academy of Sciences, Zhengzhou 450002, China; songyue@hnas.ac.cn (Y.S.); malitao@hnas.ac.cn (L.M.); duanqf@hnas.ac.cn (Q.D.); xiehf0213@hnas.ac.cn (H.X.); xinyidong@hnas.ac.cn (X.D.); zhanghr@hnas.ac.cn (H.Z.); 2 High & New Technology Research Center of Henan Academy of Sciences, Zhengzhou 450002, China; 3 School of Food Science and Engineering, South China University of Technology, Guangzhou 510640, China

**Keywords:** slow-release fertilizers, superabsorbent polymer, hydrogel, starch

## Abstract

Over the past two decades, the development and commercialization of slow-release fertilizers (SRFs) have significantly advanced, with the primary aim of mitigating environmental issues associated with excessive fertilizer use. A range of methodologies, including chemical and physical reactions, incorporation into carriers with porous and layered structures, and coating techniques, have been explored and refined. On the other hand, global challenges such as drought and desertification further underscore the need for SRFs that not only control nutrient release but also improve soil moisture retention. This paper reviews the development and application of eco-friendly starch hydrogels as fertilizer carriers and water retention for SRFs, particularly starch-based superabsorbent polymers (SAPs) produced through grafting copolymerization with acrylamide. This review explores both scientific issues, such as the microstructures and releasing mechanisms of SAPs, and technical development, involving copolymerization technologies, multi-initialization processes, methods of loading fertilizer into hydrogel, etc. Starch, as both a biodegradable and renewable carbohydrate polymer, offers distinct advantages due to its excellent chemical stability and high reactivity. The fabrication techniques of SAPs have been developed from traditional batch polymerization in aqueous solutions to more efficient, solvent-free reactive extrusion. The benefits of SRFs based on SAPs encompass enhanced soil aeration, the prevention of soil deterioration, the minimization of water evaporation, environmental pollution control, reduction in plant mortality, and prolonged nutrient retention within soil. In this review, we summarize the current progress, identify limitations in existing technologies, and propose future research directions to further enhance the performance of starch-based SRFs.

## 1. Introduction of Slow-Release Fertilizers

Data from the International Fertilizer Association (IFA) reveal a steady increase in global fertilizer consumption, reflecting the increasing reliance on chemical inputs to enhance crop yields and meet the demands of a growing global population. From 2019 to 2023, fertilizer use increased by an average of 1.3% annually [1], underscoring the agricultural sector’s continued dependence on these resources. Nitrogen fertilizers dominate the market due to their critical role in promoting plant growth by providing essential nutrients. However, their efficiency is undermined by significant losses—up to 70% of conventional fertilizers are wasted. This inefficiency has far-reaching environmental implications, contributing to pollution through decomposition, leaching, and ammonium volatilization. These processes not only deplete the quality of water resources but also contaminate soil and air, leading to a range of environmental issues such as eutrophication, soil acidification, and the emission of greenhouse gases [2,3].

To address the problems caused by overusing fertilizers, slow-release fertilizers (SRFs) have emerged as a promising solution. These fertilizers are designed to release nutrients gradually over an extended period, thereby enhancing nutrient uptake by plants and reducing the environmental impact associated with conventional fertilizers [4,5]. SRFs include a variety of products, such as controlled-release fertilizers, enhanced-efficiency fertilizers, and smart-release fertilizers, among others. Despite differences in nomenclatures, all these products should be classified as SRFs, because their primary function is to decelerate the release rate, without the ability to modulate performance based on specific crops or environmental conditions. Evaluation standards from the EU, ISO, and GB (China) also categorize these fertilizers as SRFs. Technically, there are two main strategies SRFs use to supply nutrients to plants: one involves slowing down nutrient availability in soil or reducing their solubility in water [6,7,8].

Various slow-release methodologies, including physical or chemical reactions, loading into carriers, and coating techniques, have been established [9,10,11]. Among these, coating is particularly favored for developing SRFs due to its simplicity, efficiency, and precise control over coating thickness, thereby modulating the release rate [12]. A wide range of materials have been explored as coating agents for SRFs, including petrochemical-derived polymers and biodegradable compounds like synthetic polylactic acid (PLA) and natural materials such as cellulose, lignin, starch, chitosan, and alginate [13,14,15,16]. Given concerns about potential environmental impacts from non-biodegradable materials, systems derived from biodegradable polymers, particularly those from renewable sources, have garnered significant interest. Additionally, to address challenges such as drought and desertification in agriculture, incorporating superabsorbent polymers (SAPs) into polymer-coated SRF products offers benefits such as enhanced water retention in soil and reduced soil pollution [17,18,19]. Recently, the use of eco-friendly hydrogels or SAPs as fertilizer carriers for developing SRFs has attracted growing attention [20,21]. SRFs based on SAPs offer numerous advantages, including improved soil aeration, the mitigation of soil degradation, reduced carbon emissions, and enhanced nutrient retention in soil.

While many reviews have covered SRF development from various perspectives and stages [9,22,23,24,25,26,27,28,29,30,31,32], this review focuses specifically on the use of eco-friendly hydrogels as fertilizer carriers for SRFs, emphasizing their water retention capabilities and unique release mechanisms. It addresses both scientific and technical developments, summarizing current achievements, identifying limitations in existing technologies, and proposing future research directions in this area.

## 2. Development of Starch-Based Hydrogels

Hydrogels, characterized by their unique capacity to absorb and retain large amounts of water, play a crucial role in various scientific and industrial applications. These materials are highly versatile, offering a range of properties that can be tailored to specific needs. Hydrogels consist of a network of hydrophilic polymers that are crosslinked, allowing them to maintain a solid structure despite their high water content [33]. This crosslinking is critical, as it prevents the polymer chains from dissolving in water, enabling the hydrogel to absorb water without losing its structural integrity. Both synthetic and natural polymers are used in hydrogel composition. Synthetic polymers like polyacrylic acid (PAA), polyvinyl alcohol (PVA), and polyacrylamide (PAM) are commonly used due to their controllable properties and ease of synthesis [34,35,36]. Natural polymers, such as proteins, polysaccharides, and nucleic acids, offer advantages like being bioresorbable and non-toxic, making them ideal for biomedical applications. Polysaccharides, in particular, are favored for their robustness and cost-effectiveness, with starch, alginate, chitosan, and hyaluronic acid being prominent examples. Manuel and Jennifer [37] recently conducted a comprehensive review of hydrogels, encompassing their properties, classification, synthesis mechanisms, and applications across various industries, with an emphasis on starch and cellulose as copolymer components in hydrogel formulation.

**Figure 1:**
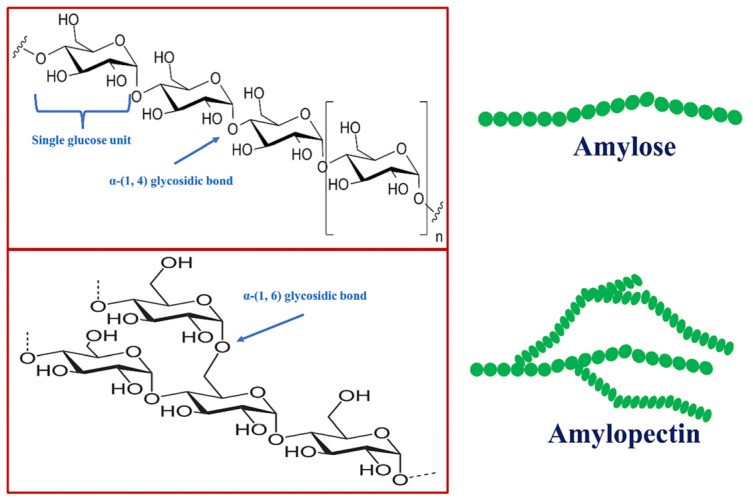
Chemical composition of starch featuring units of amylose and amylopectin.

Starch, a polysaccharide composed of amylose and amylopectin (Figure 1) [38], has high hydrophilicity, making it an attractive precursor for hydrogel synthesis. Starch-based hydrogels have garnered significant attention in agriculture due to their inherent properties such as biodegradability and eco-friendliness. However, native starch-based hydrogels face limitations, including inadequate water absorption capacity, poor mechanical stability, and limited salt tolerance [39]. To address these challenges, researchers have explored various modification techniques to enhance the properties of starch-based hydrogels [40,41,42]. One of the most effective methods is grafting, in which hydrophilic vinyl monomers such as acrylic acid (AA) and acrylamide (AM) are covalently attached to the starch backbone [43,44,45,46]. This grafting process introduces additional hydrophilic groups into the starch matrix, significantly boosting the hydrogel’s water absorption capacity and mechanical strength. Grafting is typically initiated through free radical polymerization, using chemical initiators like ammonium cerium nitrate, persulfate, and redox systems [47,48]. Crosslinking is also a critical step in hydrogel synthesis, as it determines the structural integrity and swelling behavior of the hydrogel. Various crosslinking methods have been employed, including irradiation with ultraviolet (UV) light, microwave irradiation, or gamma (γ) radiation, and chemical crosslinking agents [49,50].

Starch-based superabsorbent polymers (SAPs), known for their exceptional water absorption capability, are often synthesized via graft polymerization onto starch substrates. These SAPs can absorb several hundred to thousands of times their weight in water, making them ideal for applications such as agriculture, where they help retain water in soil and improve plant growth conditions. Additionally, SAPs can encapsulate nutrients through physicochemical interactions, allowing for controlled nutrient release, which is beneficial in slow-release fertilizer (SRF) formulations. Recent studies by Supare and Mhanwar [49] have extensively reviewed the role of SAPs in agriculture, focusing on starch-derived SAPs, crosslinking, and graft copolymerization methods used in their synthesis. This chapter delves into the methods of synthesizing starch-based SAPs under various process conditions.

### 2.1. Grafting Copolymerization with Starches

**Figure 2:**
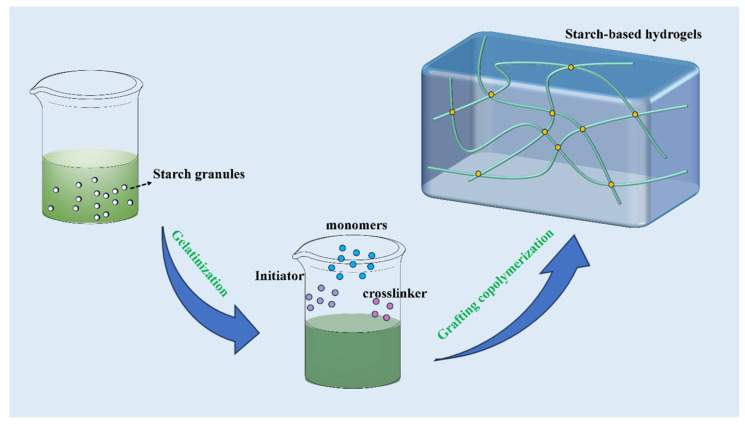
Schematic diagram of process of preparing hydrogel by starch graft polymerization in aqueous solution.

Starch-grafted PAM and PAA are among the most effective superabsorbent materials due to their superior water absorption capabilities. These materials are synthesized through graft polymerization, which typically takes place in an aqueous medium using batch processing techniques. This process enables the production of starch-based SAPs that are highly efficient in retaining water and can be used in various fields, ranging from agriculture to personal care products [51,52,53]. A schematic diagram of the process for preparing a hydrogel through starch graft polymerization in aqueous solution is shown in Figure 2. The starch is first gelatinized at a certain temperature, which destroys the crystalline structure of the starch and promotes its uniform dissolution. This step is essential, as it enhances the accessibility of starch molecules to the chemical reactions involved in grafting. Subsequently, a chemical initiator, such as a redox system, is then introduced to generate free radicals required to initiate the grafting reaction. Monomers and crosslinkers are subsequently added to the solution, and the grafting polymerization process proceeds until the desired degree of polymerization is achieved. The resulting starch-based SAPs are isolated from the reaction mixture through filtration or centrifugation, followed by washing and drying to remove any residual monomers, initiators, and by-products. The final product is a highly absorbent material capable of retaining large amounts of water, making it suitable for a wide range of applications [54,55].

Mahmoodi-Babolan et al. [56] synthesized starch-based SAPs via solution polymerization by incorporating AA and AM. The material’s performance was improved by grafting catecholamine functional groups onto its pore surfaces through dopamine oxidation polymerization. The resultant bimodal mesoporous adsorbent demonstrated a swelling ratio of 5914.66%. SAPs have also shown high efficacy as adsorbents for pollutant removal, particularly dyes, with a maximum adsorption capacity of 2276 mg/g for methylene blue.

Various starches from different resources with different microstructures have been evaluated to be used as raw materials. Zhang et al. [57] developed three types of rice starch-based superabsorbent materials (SPAMs). The findings indicated that there was a minimal occurrence of side reactions, resulting in an exceptionally high acrylic grafting efficiency of 93–94%. Substantial amylose content enhances polymer chain interactions and structural integrity through hydrogen bonding. The structured rigidity of amylopectin’s branched chains in glutinous rice starch contributes to the SPAM’s favorable water absorption properties. Siyamak et al. [58] reported on the grafting of acrylamide monomers through free radical copolymerization onto four distinct starches as potential ammonium sorbents. The findings indicated that there was no significant difference in the average monomer conversion and graft efficiency among the different types of starch. Zou et al. [59] performed an extensive investigation into the impact of the amylose–amylopectin ratio in corn-derived starches on the grafting reactions and the resultant properties of starch-based SAPs. Their results demonstrated that, under consistent reaction conditions, both the grafting ratio and efficiency increased with higher amylose content, correlating with the water absorption capacity. The high molecular weight and branched structure of amylopectin reduce polymer chain mobility, thereby increasing viscosity and potentially affecting the grafting reactions and performance attributes of starch-based SAPs. Bao et al. [60,61] investigated the relationship between molecular structures, viscoelastic properties, graft polymerization, and hydrogel microstructures using cornstarch models with varying amylose–amylopectin ratios. They found that an increase in amylose content resulted in a higher viscoelastic modulus in starch melts, which was associated with a reduced degree of micro-mixing at lower rheological dynamic rates. Although monomer conversion remained nearly constant with increasing amylose content, the grafting efficiency decreased. This decrease was attributed to the higher tendency of high-amylose starch to form crosslinks with grafted components.

In order to enhance the performance of hydrogels, particularly in terms of gel strength and water absorption capacity, researchers have been exploring the synthesis and application of composite hydrogels. One of the primary strategies in enhancing gel strength involves the introduction of reinforcing agents into the hydrogel matrix. These agents can include various types of fibers, nanoparticles, and other polymers that can increase the structural integrity of the hydrogel [62,63,64]. Li et al. [65] synthesized a starch-*g*-PAA–attapulgite superabsorbent composite by incorporating attapulgite micropowder into an aqueous reaction medium. The optimal synthesis conditions, which included 10 w% attapulgite, resulted in a composite with a water absorption capacity of 1077 g H_2_O/g sample in distilled water and 61 g H_2_O/g sample in a 0.9 w% NaCl solution. This biodegradable, cost-effective, and environmentally friendly superabsorbent composite demonstrates superior water absorbency and water retention capabilities under load, making it particularly suitable for agricultural and horticultural applications.

### 2.2. Radiation In Situ Polymerization

**Figure 3:**
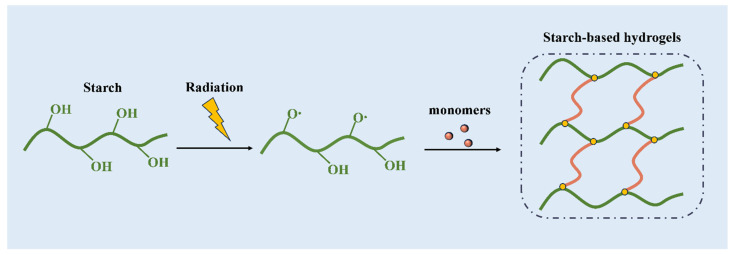
Schematic diagram of hydrogels prepared by starch irradiation graft polymerization.

Radiation technology has been extensively employed in the modification of starch, with numerous radiation techniques being utilized to produce starch-based hydrogels [66,67,68,69]. Radiations are typically classified into ionizing and nonionizing categories based on their ability to ionize materials. Ionizing radiation has the capability to ionize the medium it passes through, either through direct or indirect interaction with the material. In contrast, nonionizing radiation, due to its low ionizing potential, is incapable of ionizing materials [70]. Among ionizing radiation techniques, gamma radiation and electron beams are extensively utilized for hydrogel synthesis. Nonionizing radiation has been explored for the microwave-assisted synthesis of hydrogels [71]. As depicted in Figure 3, the radiation-induced ionization process of starch generally involves two stages. First, covalent bonds are disrupted, leading to the formation of free radicals. These radicals then initiate chemical reactions among molecules at varying concentrations [72].

Gamma irradiation has proven to be particularly effective in forming three-dimensional polymeric networks in superabsorbent materials, showcasing high efficiency [73]. This method offers several advantages over conventional hydrogel fabrication techniques. Traditional techniques often require toxic initiators and crosslinking agents, which pose risks to both human health and the environment [74]. In contrast, gamma irradiation eliminates the need for these chemicals, making the process safer and more environmentally friendly. Additionally, it allows for reactions under milder conditions, reducing energy consumption and operational costs [75]. The formation of minimal by-products further enhances the sustainability of the process by reducing waste generation and simplifies the purification steps. The irradiation process is highly controllable, allowing for precise adjustments in radiation dose to achieve the desired degree of polymerization and crosslinking [76,77]. This flexibility enables the production of starch hydrogels with tailored properties, such as absorption capacity, mechanical strength, and biodegradability [78]. Similar to gamma ray irradiation, electron beam crosslinking represents a clean and safe technique that requires no external initiators or crosslinking agents. Electron beam irradiation surpasses gamma irradiation techniques in hydrogel production, primarily due to its faster processing speed and precise beam control [79].

**Figure 4:**
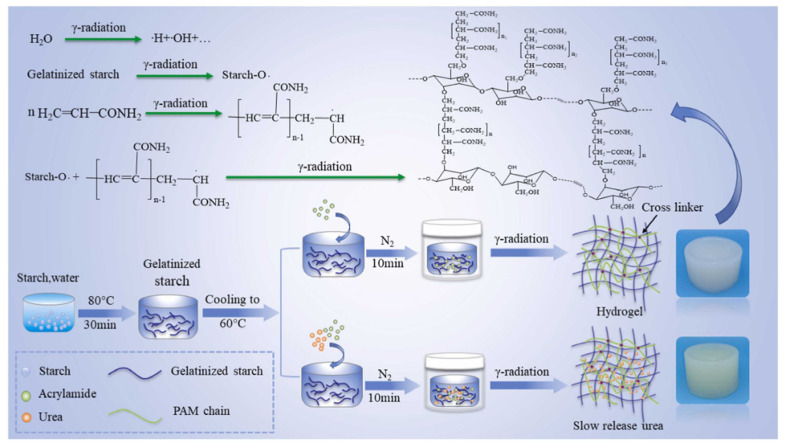
Schematic representation of procedure and principle of polyacrylamide grafted starch using γ-ray [82].

Starch-based hydrogels were synthesized at ambient temperature using gamma radiation [80,81,82], as shown in Figure 4. This study reveals that, within a certain range, increasing radiation dosage leads to a higher gel fraction. Higher doses generate more persistent free radicals, which enhances the probability of homopolymerization and the formation of an entangled network. As the radiation dose escalates, so does the concentration of free radicals, leading to a higher degree of conversion and crosslinking. However, at higher doses, intramolecular free radical recombination becomes more dominant than intermolecular recombination, particularly at sufficiently high absorbed doses, which can inhibit further crosslinking and initiate the degradation of the polymer network. When superabsorbent polymers (SAPs) were synthesized via the gamma irradiation of aqueous mixtures containing carboxymethylcellulose (CMC) and starch, the incorporation of starch significantly increased both the gel fraction and water absorption capacity at relatively low irradiation doses of 20 kGy. However, excessive starch content hindered gel formation, reducing the gel fraction. Starch is inherently prone to radiation-induced degradation, but in the presence of CMC, radicals formed on starch molecules interact with those on the CMC chains, promoting crosslinking rather than degradation [83].

### 2.3. Application of Reactive Extrusion Technology

**Figure 5:**
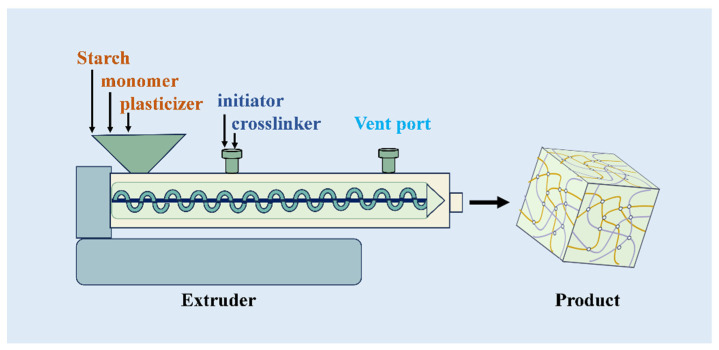
Schematic diagram of reactive extrusion to prepare starch-based materials.

Traditional methods of producing starch-based SAPs often rely on batch processes that are solvent-intensive and inefficient in terms of energy consumption and waste generation. The shift towards reactive extrusion (REX) represents a significant advancement, offering a more sustainable and efficient approach to SAP manufacturing [84,85,86,87,88]. As shown in Figure 5, REX is a continuous process that uses extruders as chemical reactors to facilitate the grafting and polymerization of starch-based materials [58]. This method is particularly advantageous for handling high-viscosity polymers, which are often difficult to process using conventional batch techniques [89]. By reducing solvent use, REX minimizes environmental impact while improving the safety and efficiency of the production process.

One of the primary advantages of REX is its operational flexibility. Various processing parameters, such as temperature and pressure, can be precisely controlled, allowing manufacturers to tailor the polymer’s properties to specific requirements [90,91,92,93,94]. Additionally, REX offers precise control over the residence time of reactants within the extruder, optimizing reaction kinetics and enhancing product performance. The continuous nature of REX leads to higher throughput rates compared to batch processes, significantly reducing production time and associated costs. This increased efficiency also enables manufacturers to respond more quickly to market demands [92]. Furthermore, the reduced need for solvents and the associated decrease in waste treatment contribute to a more sustainable manufacturing approach. Siyamak et al. [95] demonstrated the use of REX to synthesize starch-g-PAM copolymers, utilizing a screw configuration with various elements. The solvent-free graft copolymerization of starch was performed at high solid concentrations, with the reaction reaching completion within 5 min. This process resulted in an average monomer conversion rate of 80% and a grafting efficiency of approximately 74%. Another key advantage of REX is its modular design, which allows for easy modifications and optimization of the system for different types of starch-based materials and desired product characteristics [96]. This adaptability is crucial for ongoing research and development, where continuous improvements are being made to enhance SAP performance and expand their applications.

**Figure 6:**
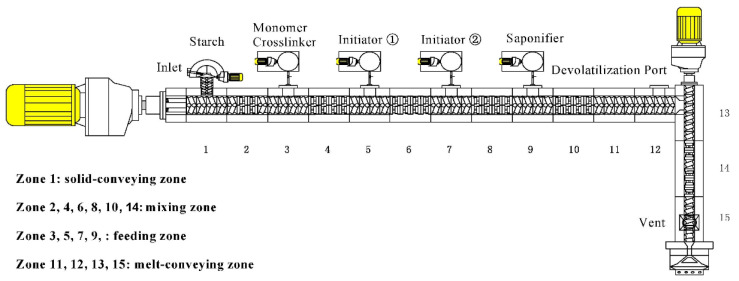
Schematic representation of dual-stage and dual-initiator reactive extrusion systems [97].

Jiang et al. [97] developed a REX system (illustrated in Figure 6) for the production of starch*-g*-PAM hydrogels, utilizing a dual-initiation mechanism. The REX system, equipped with a twin-screw co-extruder, a solid feeder, and four injection ports, facilitated the separate addition of an acrylamide solution, initiator-1, initiator-2, and a saponifying agent. The dual-initiation approach was found to enhance the homogeneity of the hydrogel’s network microstructure and augment its gel strength.

### 2.4. Characterization Techniques for Hydrogel Network Structure

Rheological Characterization of Hydrogels. Rheology is the science of studying the flow and deformation behavior of materials, aiming to investigate the response of materials under external forces [98,99]. It covers both the hydrodynamic theory of fluids and the elasticity theory of elastomers. For simple Newtonian fluids and elastomers, their rheological properties can be described by simple continuity equations such as Hooke’s law and Newton’s law of viscosity. However, the rheological properties of complex viscoelastic materials like hydrogels are much more intricate [100]. Hydrogels exhibit both viscous and elastic properties, and their behavior is influenced by stress, strain, strain rate, and even deformation history, displaying notable nonlinear characteristics [101,102]. As a typical complex viscoelastic material, the rheological characterization of starch-based hydrogels is crucial for understanding the relationship between their microstructure and macroscopic mechanical properties. Dynamic shear testing using a rotational rheometer, known for its operational simplicity and clear demonstration of viscoelastic characteristics, is the preferred technique for evaluating the viscoelastic behavior of starch-based hydrogels. This method applies periodic sinusoidal displacements and torque, measuring the response torque or strain and its phase difference from the applied action, effectively revealing the material’s viscoelastic characteristics, including storage modulus (G′), loss modulus (G″), and complex viscosity (η*). These rheological parameters not only help in understanding the interactions between starch molecular chains and the influence of crosslinking density on material performance but also guide the optimization of formulations and processing conditions to ensure product quality and meet specific application needs. (1) Strain Sweep: The linear viscoelastic region (LVR) of hydrogels can be identified through strain sweeps at constant frequency. In the low strain range, viscoelastic moduli remain independent of strain but exceed the critical strain, and the gel structure begins to break down. Chemically crosslinked gels typically have larger critical strain values than physical gels, with the LVR depending on chain interactions and crosslinking density. (2) Frequency Sweep: Frequency sweep is essential for probing the molecular structure of gel networks. By analyzing viscoelastic moduli versus frequency in a log–log plot, the relaxation behavior of gel chains is revealed. Physical crosslinking systems formed by hydrogen bonds, double helices, and ionic bonds show varied gel strengths and relaxation spectra. In highly crosslinked gels, the storage modulus (G′) is significantly higher than the loss modulus (G″) over the entire frequency range, indicating elastic behavior. (3) Time Sweep: Time sweep at constant amplitude and frequency monitors the real-time changes in the hydrogel network. It can reveal molecular structure changes and the kinetics of gel formation, with viscoelastic moduli increasing significantly during the process. (4) Temperature Sweep: Temperature sweeps involve heating or cooling gels while keeping strain and frequency constant. The intersection of G′ and G″ indicates the sol–gel transition temperature, providing insights into gel thermal stability and crosslinking strength.

Dynamic mechanical thermal analysis (DMTA) of hydrogels. DMTA is a key technique used to study the mechanical properties of materials under dynamic stress as a function of temperature or frequency [103,104]. By applying a periodic sinusoidal load to polymer-based composites and precisely recording their response, it is possible to observe a phase lag between the deformation and the applied load due to the viscoelastic nature of the material. As the temperature increases, molecular segments within the material are activated, leading to significant changes in the stiffness of the matrix. These changes can be reflected through parameters such as the storage modulus (E′), loss modulus (E″), and the loss factor (tan δ = E″/E′), which provide valuable insights for understanding and predicting the material’s performance in practical applications, particularly in assessing its viscoelasticity and energy dissipation capabilities [105].

Microstructural parameters, such as molecular weight, molecular orientation, crystallinity, the degree of crosslinking, and the nature of copolymers, significantly influence the macroscopic mechanical properties of materials. DMTA determines the glass transition temperature (*T*_g_) and secondary relaxation transitions by analyzing how E′, E″, and tan δ change with temperature. These transitions reflect changes in the material’s internal structure. For example, the glass transition temperature is the point at which a polymer transitions from a glassy state to a rubbery state, associated with the increased mobility of molecular chains. This transition directly illustrates the relationship between the material’s microstructure and its macroscopic mechanical properties. In reality, the glass transition does not occur at a single, specific temperature but over a range of temperatures where the material’s characteristics change. Various methods are used to determine *T*_g_, including the peak of the tan δ curve, the peak of the E″ curve, and the inflection point of the E’ curve. Each method has its advantages and disadvantages, and they may yield slightly different results. Typically, the temperature corresponding to the peak of the tan δ curve is taken as the *T*_g_ of the material.

## 3. Loading Fertilizer into Hydrogels

### 3.1. Coating by Starch

Among the various methods for creating SRFs, the coated and matrix types have garnered considerable attention [106]. Coated-type SRFs, in particular, have been extensively researched for their capacity to modulate nutrient release through the manipulation of coating materials [11,107,108]. These SRFs are typically prepared by applying a layer of inert material onto solid fertilizers to reduce their dissolution rate. The effectiveness of this approach heavily relies on the intrinsic physical attributes of the coating material, including its hydrophobic/hydrophilic nature and porosity. Starch granules have emerged as a promising coating material due to their ability to enhance the adhesion on fertilizer surfaces. For instance, Khan et al. [109] demonstrated a process where starch was incorporated into NPK fertilizers as a binder, ensuring the uniform coating of the NPK granules with starch particles. However, native starch has limitations such as poor film-forming ability, low water resistance, and susceptibility to damage in humid environments. To overcome these challenges, starch is often chemically modified through processes like esterification, crosslinking, and graft copolymerization to enhance its water resistance [110,111,112,113]. Modified starch as a coating material has been extensively reviewed [38], and this chapter specifically delves into the novel application of starch-based hydrogel coatings in SRFs.

**Figure 7:**
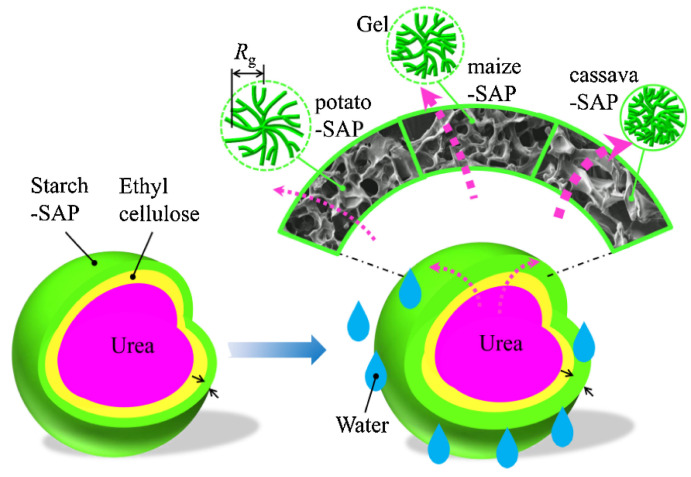
A schematic representation of the relationship between starch-based SAP characteristics and the slow-release behaviors of the double-coated fertilizer [114].

Qiao et al. [114] introduced an innovative double-coated SRF design, utilizing ethyl cellulose (EC) as the inner layer and a starch-based SAP as the outer layer, with urea as the core, accounting for 55% *w*/*w* (Figure 7). The release of nutrients from the double-coated SRF occurs in three distinct phases: (1) the absorption of water by the starch-based SAP and its subsequent permeation through the EC layer, (2) the dissolution of the urea core’s nutrients by water, and (3) nutrient delivery into soil through the dual-layered system. The release dynamics of the fertilizer are primarily influenced by the properties of the starch–SAP layer. This double-coating technique significantly augments the controlled release of nutrients and improves the overall efficiency and sustainability of fertilizer application. Similarly, Lü et al. [115] developed another dual-coated SRF that incorporated starch acetate and a combination of carboxymethyl starch and xanthan gum. By carefully selecting and modifying coating materials, researchers cancustomize the release profiles of SRFs to meet specific agricultural requirements,, ultimately leading to more effective and environmentally friendly farming practices.

Polyvinyl alcohol (PVA), a biodegradable polymer, can form a strong and environmentally friendly coating matrix when combined with starch. The enhancement in interactions and crosslinking within PVA–starch blends not only augments the strength and density of the resulting films but also impedes the swelling of the starch structure by reducing accessible regions, thereby increasing their resistance to dissolution. Zafar et al. [111] used starch and PVA coatings, acrylic acid, citric acid, and maleic acid as crosslinking agents and prepared a new type of coated urea SRF through a granulator/fluidized bed coater. The incorporation of clay nanoparticles, such as bentonite, further augments the mechanical properties of the coating film, making it more durable and effective in controlling the release of nutrients. Sarkar et al. [116] demonstrated a novel approach to encapsulate diammonium phosphate (DAP) using a blend of wheat starch, PVA, and bentonite clay, aiming to optimize the release kinetics and improve the overall performance of the fertilizer. The resulting coated DAP particles exhibited controlled release characteristics, with the release rate influenced by the concentration of bentonite clay. Higher concentrations of bentonite resulted in slower release rates, as the clay nanoparticles increased the film’s barrier properties and reduced permeability. This controlled release mechanism is beneficial for optimizing nutrient availability for plants and minimizing environmental impact by reducing nutrient leaching. This approach holds promise for the development of sustainable and efficient slow-release fertilizers, contributing to improved agricultural practices and environmental sustainability.

### 3.2. Swelling and Absorption Equilibrium Method

**Figure 8:**
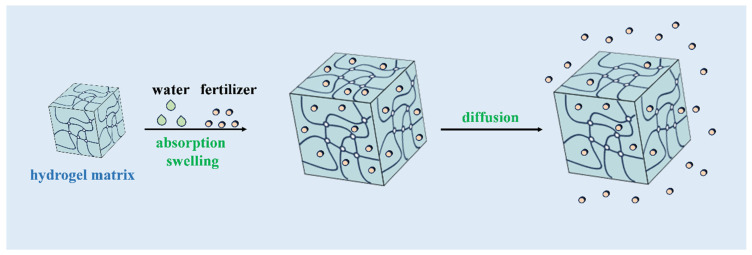
Schematic diagram of hydrogel absorption swelling and fertilizer release process.

When hydrogels are immersed in a fertilizer solution, they absorb nutrients and swell as they take up liquid. This process continues until the hydrogels reach an equilibrium state, where they have absorbed as much fertilizer solution as they can hold. These nutrient-loaded hydrogels can then be applied to soil, where they gradually release the absorbed fertilizers over time as the liquid is released (Figure 8). This method allows for the controlled and sustained release of fertilizers, improving nutrient uptake by plants and reducing the risk of nutrient leaching into the environment.

Leόn et al. [117] explored the graft copolymerization of starch with itaconic acid to produce SAPs. The findings indicated that increasing the fertilizer concentration from 0.5 g/L to 10 g/L enhanced urea absorption but reduced the adsorption of KNO_3_ and NH_4_NO_3_. The release behavior of fertilizers also varied, with higher fertilizer concentrations leading to slower release rates. Similarly, Perez et al. [118] studied macrospheres fabricated from chitosan and chitosan–starch blends. The dry polymer matrices were immersed in a fertilizer-enriched solution for 4 h at ambient temperature, then dried at 40 °C for 48 h. Analyses revealed that the bead structure significantly influenced swelling behavior, which in turn affected their fertilizer loading capacity and release kinetics. Although the swelling and absorption equilibrium method is simple, it has certain limitations. Drying the loaded hydrogels requires substantial energy, and the fertilizer loading capacity is restricted by the hydrogel’s swelling capability. Furthermore, the swelling and absorption capacity of all SAPs is significantly reduced in ionic solutions, such as those found in various fertilizers. As a result, the concentration of fertilizer within SAPs is typically modest.

### 3.3. In Situ Polymerization Technology

The in situ polymerization of starch is a method of directly initiating the polymerization of monomers in the presence of starch, which enables the formation of polymers on the surface or inside starch granules, thus improving the physicochemical properties of starch or giving it new functions [119,120]. Salimi et al. [121] fabricated a novel slow-release urea fertilizer using an in situ polymerization process. This method integrated acrylic monomers with starch in the presence of urea, achieving a homogeneous blend. The innovation was further enhanced by incorporating natural char nanoparticles as nano-fillers, which uniformly dispersed within the polymer matrix. This uniform distribution significantly improved the interfacial interactions between the polymer and the fillers, effectively retarding nitrogen diffusion and reducing the release rate in both aqueous and soil conditions.

**Figure 9:**
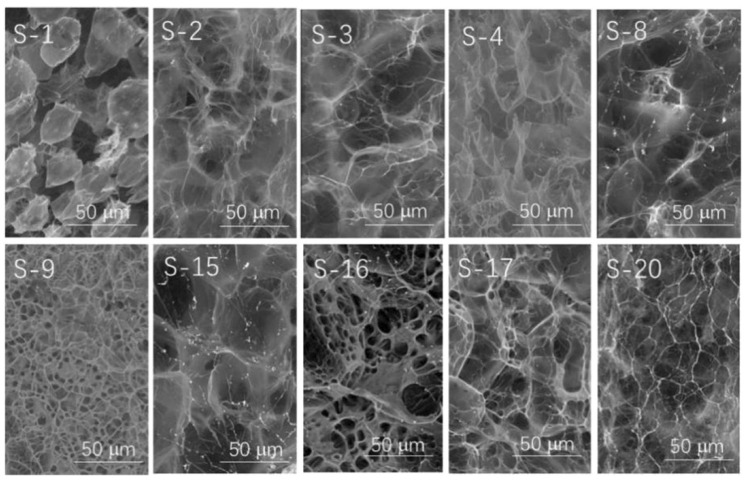
SEM images of radiation-induced in situ synthesis of starch-based hydrogels under diverse conditions. S-1–S-4, S-8: AM–starch = 1:2, water–starch = 10:1, irradiation doses are 3 kGy, 6 kGy, 9 kGy, 12 kGy, and 24 kGy, respectively. S-9: AM–starch = 1:2, water–starch = 6:1; S-15: AM–starch = 1:2, water–starch = 15:1; S-16: AM–starch = 2:1, water–starch = 10:1; S-17: AM–starch = 2:2, water–starch = 10:1; S-20: AM–starch = 2:6, water–starch = 10:1, irradiation dose is similarly 12 kGy [82].

Chen et al. [82] developed an approach for synthesizing SRFs through the in situ radiation-induced polymerization of monolithic hydrogels. This method involves embedding urea within a starch-based matrix and grafting it with polyacrylamide. The analysis demonstrated distinct microstructures in hydrogels prepared under different conditions (Figure 9). Low irradiation intensities preserved some honeycomb structures with weaker fibers, but higher intensities resulted in the disappearance of these structures, replaced by larger holes and a denser, more homogeneous crosslinked network. Higher concentrations led to porous structures, whereas lower concentrations produced less dense networks and thinner cell walls. The presence of porous or cottony structures was primarily attributed to the grafted starch matrix, emphasizing the significant influence of irradiation intensity, concentration, and the AM–starch ratio on the hydrogel microstructure. An increase in radiation intensity and concentration enhanced grafting efficiency and monomer conversion. Enhanced gel strength, associated with higher radiation levels, AM content, and concentration, was correlated with a reduced urea release rate. For example, the release rates of samples U-1~U-7 were only about 26.2%, 14.9%, 20.2%, 11.6%, 33.5%, 9.5%, and 30.2% in 2 h, respectively. After 42 h, the release rate of urea remained above 80%, and the difference was not significant.

### 3.4. Reactive Extrusion Technology

REX is a continuous process that involves the mixing and chemical modification of polymers in the molten state within an extruder. The extruder is a long, cylindrical machine equipped with a screw that rotates and conveys the polymer forward while mixing it with other components [122]. The REX process for loading fertilizer into hydrogels typically involves the following steps: (1) Preparation of hydrogel precursors. The first step is the preparation of hydrogel precursors, which are usually hydrophilic polymers such as PVA, PAA, PAM, starch, cellulose, etc. (2) Incorporation of fertilizer. The fertilizer, which can be in the form of solid particles or a liquid solution, is introduced into the extruder along with the hydrogel precursors. The extruder is designed to provide a controlled environment for the mixing and encapsulation of the fertilizer within the polymer matrix. (3) Crosslinking reaction. As the mixture moves along the extruder, a crosslinking reaction is initiated, typically through the addition of a crosslinking agent or by using heat or radiation. This reaction leads to the formation of the hydrogel network, with the fertilizer particles or molecules being trapped within the polymer matrix. (4) Formation of hydrogel beads. The hydrogel–fertilizer mixture is then extruded through a die, typically in the form of small beads or pellets. These beads are rapidly cooled to solidify the hydrogel structure and stabilize the encapsulated fertilizer.

REX research encompasses a broad spectrum of disciplines, such as chemical reaction engineering, rheology, polymer processing, and mechanics, involving numerous intricate reaction processes. Given the complexity of these processes, the utilization of reactive extrusion in the production of starch-based SRFs remains relatively constrained. A modified Hakke internal mixer was employed as a reactor to synthesize SAPs, mimicking the operation of a twin-screw extruder [60,61,123,124,125]; Under a high-viscosity system, the fertilizer loading rate can be increased to over 37 w% [126]. The REX process, while efficient for many polymer processing applications, presents a unique challenge when it comes to the preservation of the crosslinked structure of hydrogels. The crosslinking in hydrogels is a critical factor that determines their mechanical strength, swelling behavior, and degradation rate. During the reaction extrusion process, which involves the extrusion of a polymer melt through a die, the high shear forces and thermal conditions can potentially disrupt the delicate crosslinked structure of the hydrogel. This disruption can lead to a decrease in the mechanical integrity of the hydrogel, affecting its performance in practical applications. To circumvent the potential degradation of the crosslinked structure during the reaction extrusion process, an alternative approach known as post-crosslinking can be employed. Post-crosslinking involves the formation of crosslinks in a polymer matrix after the initial processing steps. This method allows for the preparation of hydrogels with controlled crosslinking density and distribution, which is crucial for optimizing the properties of the hydrogel for specific applications.

### 3.5. Compounding with Cellulose

Cellulose, a polymeric compound abundantly found in plant cell walls, is the most plentiful and sustainably sourced material on Earth. It possesses a rich variety of functional groups, exceptional mechanical properties, and a high degree of chemical adaptability, making it ideal for modification [127,128]. Furthermore, the capability to render cellulose into fibrous forms at the micro- or nanometer scale has positioned it as an increasingly promising contender for the creation of hydrogels in recent years. Li et al. [129] reviewed various modification methods of cellulose and the application of stimuli-responsive hydrogels in slow-release fertilizers. Manuel et al. [37] conducted a review on hydrogels, delving into their properties, classifications, synthesis mechanisms, and diverse applications across various industries. In their article, starch and cellulose were highlighted as copolymers, showcasing their integral roles in the development and functionality of hydrogels.

**Figure 10:**
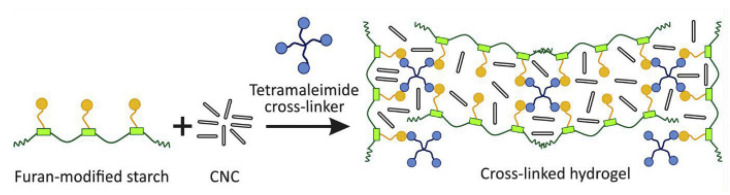
Illustrative scheme of structure of cellulose nanocrystals compounded with starch-based hydrogel [130].

Cellulose-based composites have garnered significant attention in recent years due to their potential as reinforcing fillers in biopolymer matrices derived from starch [64,130,131,132] (Figure 10). Starch composite cellulose hydrogels are both biodegradable and renewable polymers, and these hydrogels can slowly release nutrients to plants, providing a controlled and sustained supply of essential elements for plant growth. Bora et al. [133] utilized wastepaper powder as a modifier in a biodegradable hydrogel composite composed of starch, itaconic acid, and acrylic acid. The incorporation of an optimal quantity of the modifier increased the hydrogel’s swelling capacity from 503 g/g to 647 g/g. The NPK-laden hydrogel demonstrated effective sustained-release characteristics, with 98% of nitrogen, 81% of phosphorus, and 95% of potassium being released over 20 days.

### 3.6. Other Hydrogel Systems

Interpenetrating polymer network (IPN) hydrogels based on starch represent a promising class of materials that combine the biodegradability and biocompatibility of starch with the mechanical properties and functional versatility of synthetic polymers. These hydrogels are formed by the simultaneous or sequential polymerization of starch with one or more synthetic polymers, resulting in a network structure where both components are interwoven. This interpenetration enhances the overall performance of the hydrogel, rendering it appropriate for SRFs. Vudjung et al. explored the synthesis of crosslinked natural rubber and cassava starch hydrogels through the IPN method [134]. This approach aimed to enhance mechanical strength and compatibility by integrating multiple network polymers. These hydrogels demonstrated high water swelling capabilities and favorable biodegradation properties. However, the rigid nature of the final product precluded its application as a coating on urea bead surfaces. To mitigate this issue, they sought to innovate it by developing a novel coating membrane from pre-vulcanized natural rubber (NR) and starch (St), employing the IPN method with sulfur and glutaraldehyde as crosslinking agents [135]. The release mechanism of urea in both aqueous and soil environments was characterized as non-Fickian diffusion, suggesting transport through a porous matrix. The efficacy of encapsulated urea beads in corn and basil cultivation was significantly superior to that of native urea beads.

Apart from cellulose, starch can also form complexes with other polysaccharides to construct an SRF system. Pimsen et al. [136] focused on the creation of SRFs utilizing a nano-zeolite (NZ) composite incorporated into chitosan (CS)–sago starch (ST)-based biopolymers. The biopolymer nanocomposite was synthesized through an ionotropic gelation method, employing sodium tripolyphosphate as the crosslinking agent. The swelling capacity of the biopolymer nanocomposite notably increased with higher molecular weights of CS and increased crosslinking durations. The NZ-CS/ST nanocomposite exhibited a release of 64.00% phosphorus and 41.93% urea by the 14th day. Majeed et al. [137] investigated the impact of lignin concentration on the release and biodegradability of urea-modified tapioca starch-based enhanced-efficiency fertilizers. They found that incorporating lignin at levels of 5%, 10%, 15%, and 20% into the starch matrix decreased nutrient release in moist soil. Furthermore, an increase in lignin content was associated with enhanced biodegradability.

The integration of inorganic fillers into starch-based hydrogel SRFs represents a significant advancement in agricultural technology, offering multifaceted benefits that enhance the overall efficacy and sustainability of fertilizer applications. This integration is particularly crucial in modern agriculture, where the need for efficient, environmentally friendly, and cost-effective fertilization methods is paramount. These fillers serve as potent reinforcing agents, significantly bolstering the hydrogel’s resilience against mechanical stress, including wear and compression [138]. Furthermore, they enhance the hydrogel’s capacity to retain water, ensuring a robust performance in various applications. Lu et al. [139] studied a novel slow-release fertilizer characterized by high water retention, designated as HS-BCF, which was synthesized by integrating hydrotalcite and starch into biochar-based compound fertilizers (BCFs). The findings indicate that the addition of hydrotalcite and starch to BCFs enhanced the soil water retention capacity by 5–10%. Over 30 days, the cumulative leaching amounts of nitrogen, phosphorus, and potassium from HS-BCF in soil were, at most, 49.4%, 13.3%, and 87.4% of those from BCFs, respectively. Furthermore, hydrotalcite was found to bind with phosphorus in HS-BCF, thereby improving the longevity of phosphorus in the fertilizer. Wei et al. [140] reported on a novel slow-release and water retention fertilizer through the free radical copolymerization of potato starch, acrylic acid, acrylamide, and maleic anhydride-modified β-cyclodextrin. This synthesis process was enhanced by incorporating acid-treated halloysite nanotubes, which were engineered to expand their internal cavities for optimal urea pre-loading. The addition of these halloysite nanotubes improved the fertilizer’s release profile, effectively regulating the cumulative release rate of urea from the slow-release formulation.

Starch-based hydrogels SRFs have been extensively studied, with numerous research findings presented in Table 1.

## 4. Release Mechanisms and Kinetics

### 4.1. Release Mechanisms

The release of nutrients from SRF hydrogels is a complex, multi-stage process involving various mechanisms. A comprehensive understanding of these mechanisms is essential for optimizing the design and application of hydrogels in agriculture. Nutrient release typically follows three distinct phases, the lag phase, the constant release phase, and the decay period, each contributing uniquely to the overall release dynamics [150]. During the lag phase, the hydrogel absorbs water from the surrounding environment, initiating the swelling process. This initial stage is characterized by minimal nutrient release, as the hydrogel primarily undergoes hydration and expansion. Following this is the constant release stage, during which the hydrogel reaches an equilibrium state, and nutrients are steadily released. This stage is driven by the diffusion of nutrients through the polymer matrix and the continued hydration and expansion of polymer chains. The decay phase occurs when the release rate of nutrients begins to decline, typically due to the depletion of available nutrients within the hydrogel or changes in environmental conditions.

Release mechanisms involve multiple processes, including diffusion through the polymer and the hydration, expansion, and dissolution of polymer chains [151,152,153]. Diffusion through the polymer matrix is critical, enabling nutrients to diffuse from higher to lower concentration regions. This process is influenced by the polymer network, nutrient molecule size, and the crosslinking degree. The hydration and expansion of polymer chains also play an essential role. As the hydrogel absorbs water, the polymer swells, creating additional space within the polymer matrix, which facilitates nutrient movement and release into the environment. The degree of swelling depends on factors like polymer hydrophilicity, crosslinking, and environmental conditions such as ionic strength and pH. Polymers with higher hydrophilicity and lower crosslinking density tend to exhibit greater swelling, enhancing nutrient release.

Various kinetic models have been developed to understand how swelling impacts nutrient release [154,155]. Swelling behavior occurs in two stages: an initial rapid swelling phase, followed by a gradual and slower swelling phase. In the initial phase, water rapidly penetrates the hydrogel’s porous matrix, driven by the polymer’s hydrophilic nature. In the gradual swelling phase, water penetrates the pores induced by the relaxation of polymer chains. This process is slower and more protracted, reflecting the gradual uptake of water and the continued expansion of the polymer matrix. Polymers with higher hydrophilicity and lower crosslinking tend to exhibit more rapid swelling, while higher crosslinking density and lower hydrophilicity result in slower, more gradual swelling.

Environmental factors, such as temperature, pH, and ionic strength, significantly affect swelling and, consequently, nutrient release. For instance, higher temperatures enhance the diffusion of water into the hydrogel matrix, speeding up swelling and nutrient delivery. Changes in pH can affect the ionization of functional groups, altering the swelling behavior of the hydrogel. Ionic strength can affect osmotic pressure, which drives water absorption [156,157]. Dudu et al. [144] synthesized a novel superabsorbent based on N,N-dimethylacrylamide, maleic acid (MA), and starch (St), designated as DMSt_1_, and evaluated its performance as a controlled-release fertilizer in lettuce cultivation. They subjected DMSt_1_ to surface modifications using HCl and NaOH, resulting in negatively and positively charged variants, DMSt_2_ and DMSt_3_, respectively. The latter exhibited the highest maximum swelling rate of 37.38% in deionized water. The acid and base treatments altered the anionic and cationic properties of DMSt_1_, leading to distinct swelling behaviors at varying pH levels. The hydrogels derived from DMSt_1_, DMSt_2_, and DMSt_3_ demonstrated remarkable water uptake capacities, with equilibrium swelling values of 7163.7% at pH 10, 15708% at pH 10, and 27838% at pH 8, respectively. de Lima et al. [158] developed a hydrogel through the copolymerization of cassava gum and polyacrylamide. The hydrogel exhibited the maximum swelling at pH 7, absorbing approximately 270 times its initial mass. At pHs 4 and 2, the swelling capacity decreased to 150 and 250 times, respectively. This reduction in swelling at lower pH values is attributed to the protonation of carboxylate anions, which diminishes repulsive forces and impedes swelling. In more alkaline environments, the swelling percentage also decreased, reaching 100 at pH 10. This phenomenon is explained by the “charge shielding effect”, which weakens electrostatic interactions, as excess Na^+^ ions hinder the formation of simple COO bonds, a consequence of the hydrolysis process. Al Rohily et al. [159] investigated the swelling properties of fertilizer materials derived from phosphorylated alginate grafted with polyacrylamide (PAM). They found that the degree of crosslinking and the presence of pendant ionic groups within the polymer matrix significantly influence the swelling behavior. In these hydrogels, ionic charges play a key role, as the repulsion between adjacent fixed charges causes network expansion and subsequent swelling. Shang et al. [154] used the Korsmeyer–Peppas and pseudo-second-order kinetic models to analyze the swelling characteristics of temperature-responsive hydrogels, concluding that water transport is primarily governed by Fickian diffusion and polymer chain relaxation.

Overall, the release of nutrients from SRF hydrogels involves a combination of diffusion, swelling and deswelling, chemical degradation, mechanical stress, ion exchange, and temperature effects. These mechanisms work synergistically to provide a controlled and sustained release of nutrients to plants, enhancing nutrient use efficiency and promoting plant growth. A clear understanding of these mechanisms and their interaction with environmental factors is essential for designing effective hydrogel-based fertilizers.

### 4.2. Kinetic Models

The development of release kinetic models for fertilizers plays a critical role in agricultural science, particularly optimizing nutrient delivery to plants. These models are essential for understanding and predicting the behavior of fertilizers under various environmental conditions, aiding in maximizing crop yields while minimizing environmental impact. Quantitative analysis is vital for developing these models because nutrient release is dynamic—fertilizers do not release nutrients at a constant rate. Instead, the release rate can vary significantly depending on factors such as temperature, moisture, and soil biological activity. Quantitative analysis allows for the measurement of these variables over time, providing data that can be used to construct mathematical models that accurately predict nutrient release patterns. For instance, the type of fertilizer, whether a conventional, slow-release, or controlled-release formulation, significantly impacts the release kinetics. Given the variability in nutrient release mechanisms among various types of SRFs and the intricate interplay of factors such as composition, soil moisture, and temperature, the release mechanisms of SRFs cannot be simply characterized. Typically, four primary mathematical models are employed to elucidate these mechanisms: the zero-order, first-order, Korsmeyer–Peppas, and Higuchi kinetic models can be represented by the following equations (Equations (1)–(4), respectively):

Zero-order model: M_t_/M_∞_ = k_0_t (1)

First-order model: M_t_/M_∞_ = 1 − e(−k_1_t) (2)

Korsmeyer–Peppas model: M_t_/M_∞_ = kt^n^ (3)

Higuchi model: M_t_/M_∞_ = k_H_t^1/2^ (4)

Specifically, the parameter M_t_/M_∞_ denotes the percentage of fertilizer released from the hydrogel at a specific time t. The constants k_0_, k_1_, k_H_, and k represent the release constants for the zero-order, first-order, Higuchi, and Korsmeyer–Peppas models, respectively. The release mechanisms are classified according to the diffusion index (n) as follows: (1) Fickian diffusion mechanism: n < 0.43; (2) non-Fickian diffusion mechanism: 0.43 < n < 0.85; and (3) Case-II transport mechanism: n > 0.85 [129].

Indeed, the slow-release model of the SRF hydrogel exhibits variation across different stages. Shaghaleh et al. [160] developed an aminated cellulose nanofiber (A-CNF) fertilizer hydrogel by encapsulating ammonium nitrate (AN) within the A-CNF matrix. During the initial phase, the hydrogel demonstrated rapid AN release within the first 72 h, conforming to a first-order kinetic model (k_1_ = 0.068–0.0575). This phase involved the swelling of A-CNF when exposed to buffer or soil media, where pH levels influenced AN diffusion. In the subsequent phase, spanning 144 to 504 h, the hydrogel followed a zero-order kinetic mechanism, demonstrating a controlled release with a lower AN release rate (k_0_ = 0.0023–0.0027). The final release phase, occurring between 720 and 1540 h, involved the degradation of the hydrogel’s polymeric network, particularly in soil conditions. This degradation resulted in the breakdown of A-CNFs and the formation of oligomeric fragments, enlarging the pore structure and enhancing AN release, which was previously restricted within the dense hydrogel matrix. This stage followed the Higuchi model, with an increased release rate (k_H_ = 0.0281–0.0268). Notably, soil properties significantly moderated the AN release rates across all stages and pH levels, compared to buffer media, leading to a more consistent and stable AN release profile.

Water permeation and the subsequent chain relaxation in hydrogels are pivotal in determining swelling dynamics and nutrient release kinetics. Tanan et al. [141] developed biodegradable semi-IPN hydrogels composed of cassava starch (CSt)-g-PAA, natural rubber (NR), and PVA. Urea release in both aqueous and soil environments conformed to the Korsmeyer–Peppas model. When NR content was ≤50%, the behavior was non-Fickian, with both water diffusion and polymer chain relaxation governing the swelling process. When NR content exceeded 50%, diffusion was pseudo-Fickian (n < 0.5), indicating that hydrophobicity rather than chain relaxation primarily controlled water penetration, leading to a lower equilibrium swelling ratio (S_eq_). These findings show that S_eq_ and n values can be adjusted by altering the NR/PVA ratio to meet specific application needs. Chamorro et al. [161] prepared cassava starch hydrogels in an aqueous environment. Upon swelling, the polymer matrix undergoes macromolecular relaxation, transitioning to a rubbery state that typically allows for solute diffusion into the surrounding aqueous phase. However, if the rate of water penetration is significantly lower than the polymer chain relaxation rate, the diffusion is characterized as “less Fickian” with an “n” value less than 0.5, although it remains within the Fickian classification. The release kinetics of potassium followed a less Fickian diffusion pattern, suggesting that its release is predominantly governed by the water intake rate, which promotes swift diffusion through the hydrogel’s rubbery matrix. Nitrogen release, on the other hand, was influenced by the hydrogel’s high fluidity, which facilitated water permeation into the biopolymer network. The release kinetic models mentioned above are the most widely used in the study of hydrogel SRFs, but due to the complexity of the fertilizer release process, other kinetic models have been studied and applied, such as the Peppas–Sahlin, Hixson–Crowell, and Ritger–Peppas models [150,158,162,163].

In conclusion, release kinetic models are indispensable tools in agricultural science. They provide a quantitative framework for understanding and predicting fertilizer behavior, which is crucial for optimizing nutrient delivery to plants and ensuring sustainable agricultural practices. By combining quantitative analysis with mathematical and geometrical models, researchers can develop more effective and environmentally friendly fertilizer formulations, ultimately leading to healthier plants and higher crop yields

## 5. Conclusions and Future Directions

In the pursuit of sustainable agricultural practices, the development of controlled-release fertilizers (CRFs) and smart fertilizers has garnered significant attention. These innovative fertilizers are designed to optimize nutrient delivery to crops in a manner that closely aligns with their growth requirements, thereby enhancing plant health and productivity while minimizing environmental impacts. The use of eco-friendly starch hydrogels in the formulation of these fertilizers not only aids in slow-release mechanisms but also contributes to improved water retention in soil, a critical factor in arid and semi-arid regions where water scarcity is a pressing issue. Starch hydrogels, derived from renewable plant sources, offer a biodegradable and environmentally friendly alternative to traditional synthetic polymers used in fertilizer encapsulation. These hydrogels can be engineered to have varying degrees of permeability, allowing for precise control over the release of nutrients into soil. The incorporation of starch hydrogels into fertilizer formulations can significantly reduce the initial burst effect commonly associated with conventional fertilizers, where a large amount of nutrients is released quickly, often leading to nutrient leaching and runoff.

**Figure 11:**
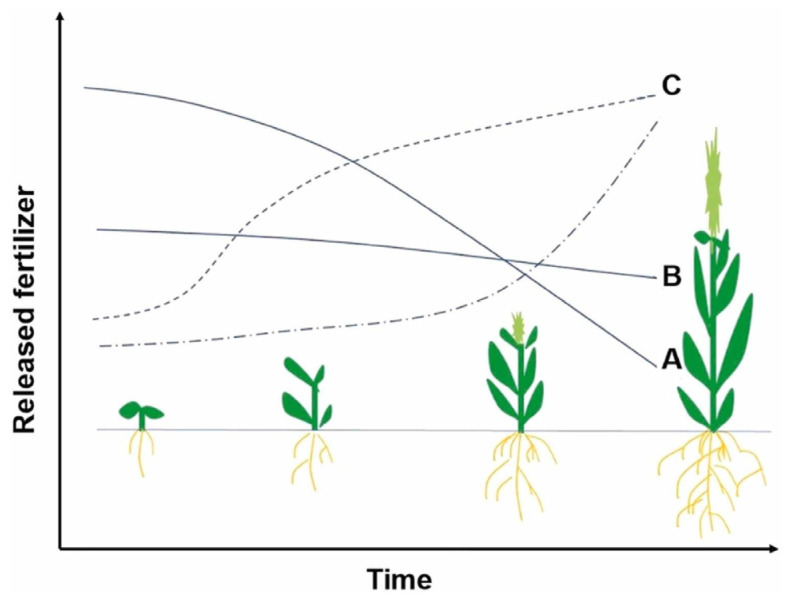
Schematic representation of plant growth vs. released fertilizer for different fertilizers: A: raw fertilizer; B: slow-release fertilizer; C: controlled or smart fertilizer [9].

Figure 11 provides a schematic depiction of plant growth in relation to the release of fertilizer, highlighting the discrepancy between the conventional rates of fertilizer release and the nutrient requirements of plants [164,165]. Corn, for instance, undergoes distinct growth phases, sowing, seedling, tasseling, and grain filling, each with varying nutrient demands. Traditional fertilizers like urea, due to their high solubility, release nutrients rapidly, often at times when the plant requires them the least. This mismatch can lead to inefficiencies in nutrient utilization and potential environmental harm.

Smart fertilizers, on the other hand, are designed to release nutrients in a controlled manner that mimics the natural uptake patterns of plants. By adjusting the composition and structure of starch hydrogels, researchers can modulate the release kinetics of nutrients, ensuring that they are available to the plant when needed. This approach not only enhances crop yields but also reduces the overall amount of fertilizer required, making agriculture more sustainable and cost-effective. The potential of smart fertilizers extends beyond traditional soil-based agriculture to soilless cultures, including hydroponics and aeroponics. In these systems, precise control over nutrient delivery is crucial for maintaining optimal plant growth conditions. Starch hydrogels can be adapted for use in nutrient reservoirs, providing a steady and controlled release of nutrients directly to plant roots. This application can lead to more efficient nutrient use and reduced waste, further supporting the sustainability of soilless cultivation methods.

Despite promising developments in the field of smart fertilizers, several challenges remain. The development of truly responsive fertilizers that can adapt to changing environmental conditions and plant needs is still in its infancy. Future research should focus on integrating advanced sensing technologies with fertilizer formulations to create fertilizers that can dynamically adjust their release rates based on real-time data on soil conditions, weather patterns, and plant health. Moreover, the commercialization of these advanced fertilizers requires a careful consideration of cost-effectiveness and scalability. While the benefits of using starch hydrogels and other eco-friendly materials are clear, their adoption will depend on their economic viability compared to traditional fertilizers.

The development of slow-releasing fertilizers with water retention capabilities using eco-friendly starch hydrogels represents a significant step forward in sustainable agriculture. By aligning nutrient release with plant growth needs and enhancing soil moisture retention, these fertilizers can contribute to more efficient and environmentally friendly farming practices. As research continues to advance, the potential for these smart fertilizers to revolutionize agriculture and support global food security becomes increasingly evident.

## Figures and Tables

**Table 1:** Summary of starch-based SRF hydrogels.

Starch/Additives	Fertilizer	Modification	Performance	References
Cassava starch–PAA/natural rubber/PVA	Urea	CSt-g-PAA networks and linear NR/PVA blends	In water: hydrogel wax coated urea (BHWCU) <60% within 24 h, in 7 days, 89.1%, 68.6%, and 41.5% in BHWCU/2:8, BHWCU/6:4, and BHWCU/9:1 formulations. In soil: hydrogel wax coated urea <68% in 20 days	[141]
Cassava starch-g-poly(acrylonitrile)	Urea	Crosslinking of cassava starch and acrylonitrile using MBA applied as a coating	Uncoated urea released 100% after 28 days in soil, coated urea 70% in 108 days	[15]
Corn starch–Poly (AA-co-AM)	Diammonium phosphate	Crosslinking of starch, AA, and AM using MBA	60% N release during 30 days in soil	[142]
High-amylose maize starch-g-poly(sodium acid maleate-disodium maleate)	Potassium	High-amylose maize starch graft copolymerization with disodium maleate, using KMnO_4_-NaHSO_3_ redox system, and covalently crosslinked with MBA	The release percentages of KNO_3_ and KH_2_PO_4_ were between 92 and 67% and 89 and 61% in 320 h	[143]
Poly (N, N-dimethylacrylamide –maleic acid)–starch	Urea	Crosslinking of starch, N, N-dimethylacrylamide, and maleic acid using MBA	Urea released from hydrogels DMSt1, DMSt2, and DMSt3 after 14,000 min was found as 80.2%, 45.5%, and 67.7% in well water and 81.5%, 46.6%, and 68.9% in tap water	[144]
Sodium alginate–carboxymethyl starch sodium–polydopamine	Urea	Sodium alginate and carboxymethyl starch sodium were compounded, and polydopamine (PDA) film was formed on its surface by self-polymerization	In soil: >25 days	[145]
Corn starch–castor oil superabsorbent–polyurethane	Urea	Starch–castor oil SAP–polyurethane-coated urea in smooth rotating drum	Nitrogen-controlled release period of 60–150 days in water	[146]
Starch carbamate–sodium alginate–SRF	Urea	Starch carbamate and sodium alginate through cationic ion crosslinking	In water: 61.6% within 10 h and almost completely release >16 h. In soil: 58.5% of urea released within 25 days and exceeded 50 days for complete release	[147]
Starch phosphate carbamate–PVA–stearic acid	Urea	Stearic acid as inner coating layer and starch phosphate carbamate–PVA film crosslinked with citric acid as external layer	In water: 50.3% within 15 h, nearly complete release over 25 h. In soil: 46.6% was released within 20 d, extending to approximately 30 d	[148]
Chitosan–starch	Urea	Crosslinking of starch and chitosan using glutaraldehyde	63.71% N release during seven days in soil	[149]

## Data Availability

Not applicable.
